# Case series report on long-term result of endovascular approach to thrombosed limb or limb graft occlusion of aortoiliac endoprosthetic stent graft using the first-order percutaneous mechanical arterial advanced thrombectomy technology protocol

**DOI:** 10.1016/j.jvscit.2025.102071

**Published:** 2025-11-27

**Authors:** Alexander Cartwright, Ellie Gamradt, Brayan Marino, Ambika Singh, Beckett Peterson, David Peterson, Duangnapa Cuddy

**Affiliations:** a Indiana University School of Medicine, Bloomington, IN; b University of Minnesota Medical School, Duluth, MN; c NSU Florida, Dr. Kiran C. Patel College of Osteopathic Medicine, Ft. Lauderdale, FL; d University of Michigan, Ann Arbor, MI; e Indiana University Health, Bloomington, IN

**Keywords:** Limb graft occlusion after endovascular aortic aneurysm repair, Aortoiliac endoprosthetic limb graft occlusion, Percutaneous repair, FOPMAATT protocol

## Abstract

To address the limitations of conventional management of limb graft occlusion after endovascular aortic aneurysm repair and endoprosthetic stent graft placement for aortoiliac occlusive disease, our team pioneered an innovative technique known as first-order percutaneous mechanical arterial advanced thrombectomy technology (FOPMAATT). We previously reported a single case where the benefits of the technique were demonstrated in 2022 by Willhite et al. The case report described the use of the FOPMAATT protocol in a patient with limb graft occlusion after EVAR endoprosthetic stent graft implantation for abdominal aortic aneurysm endovascular repair. In this consecutive case series, five patients underwent the FOPMAATT protocol procedure, resulting in positive patient outcomes such as a low postoperative complication rate. Each patient was followed for ≥2 years to assess long-term outcomes. Of the five cases reported, we did not observe a significant adverse perioperative event, no blood loss significant enough to require transfusion, or length of stay of >1 day owing to any surgical cause. Additionally, we did not use adjuvant pharmacological thrombolytic therapy in the FOPMAATT protocol, which may decrease the risk of perioperative bleeding.

Within the first 6 months after an endovascular aortic aneurysm repair (EVAR), iliac arterial limb graft occlusion (LGO) is among the leading causes of reintervention.^1^^,^^2^ With an incidence rate of 1% to 6%, LGO remains an under-reported complication of EVAR.^3^ Additionally, LGO is also a well-recognized complication of aortoiliac stent graft revascularization for occlusive disease.

After EVAR, LGO may happen in one of the limbs or both from different etiologies. An endoprosthetic stent graft placed within the aortoiliac bifurcation as a kissing stent for aortoiliac occlusive disease may also have a complication causing one or both of the limbs to become occluded or thrombosed (LGO) from different etiologies. Both complications present real and related problems for patients.^1^^,^^2^

The risk of an LGO may be attributed to patient compliance (eg, tobacco use), anatomical factors, device-related factors, including device type, or a combination thereof.^4^ Treatment for LGO includes open surgery and endovascular techniques that allow for bypass, thrombolysis, and thrombectomy.^1^ Risks associated with open surgical repair and operative femoral cutdown include increased blood loss, postoperative wound complications, and longer hospital stays. Catheter-directed pharmacological thrombolytic therapy may also increase risk of bleeding, although it may offer a percutaneous therapeutic approach.^5^, ^6^ As a result, the current disadvantages of LGO management have initiated the pursuit of innovative treatment options.^2^^,^^3^^,^^7^

In our previous study, we reported a novel endovascular approach that has been proven to decrease mortality, length of hospital stay, and morbidity in the setting of symptomatic thrombosis of the left iliac limb after EVAR.^8^ This case series reports the long-term patient outcomes of the continued use of a novel endovascular approach in the treatment of aortoiliac LGO after EVAR for AAA and after aortoiliac endoprosthetic stent graft for occlusive disease over the course of ≥24 months. The study was performed at the Indiana University Hospital, Regional Academic Health Center in Bloomington, which serves the rural population of Southern Indiana. Compared with existing interventions, the reported approach may help to decrease morbidity with fewer associated wound healing complications and limited blood loss. As a result, patients may experience a shorter hospital stay, and the protocol may result in an overall decrease in treatment cost.

## Methods

This is a report of five consecutive cases at a single institution. In the first case, the patient experienced thrombosed endoprosthetic stent graft within 3 months after endovascular repair of AAA. The subsequent consecutive four cases, patients experienced thrombotic occlusive endoprosthetic stent graft complications after aortoiliac occlusive disease endovascular revascularization. All five cases were managed successfully with the novel first-order percutaneous mechanical arterial advanced thrombectomy technology (FOPMAATT) protocol technique, which allowed for a totally percutaneous approach. The purpose of this case series report was to demonstrate the results of surgical management using FOPMAATT protocol within a single institution. All patients provided consent for the collection and publication of their data. Indiana University Human Research Protection Program (HRPP) approved this medical educational endeavour (#19902).

### FOPMAATT protocol

This section is adapted from Willhite et al.^8^ This technique requires a minimum patency of 4 cm for a common femoral artery ipsilateral access site, which is the affected side (Fig). This is the patent common femoral artery length needed to accommodate the funnel tip of the sheath for percutaneous embolic protection. This may be considered as an exclusion criterion for the patient to undergo FOPMAATT protocol management. Within this protocol description, the authors use the term ipsilateral to describe the thrombosed/occlusive limb graft side and the term contralateral to describe the nonaffected side. Distal embolization control is achieved by using the built-in nitinol funnel-shaped sheath at the ipsilateral side access. Currently, there is no first-order branch percutaneous arterial thrombectomy device that allows retrograde access with a built-in embolic protection mechanism. A known conventional percutaneous catheter-directed thrombolytic therapy may allow intravascular ultrasound (IVUS) interrogation at the subsequent stage of the procedure. However, the technique may increase the risk of bleeding with thrombolytic infusion, additional hospital stay, and difficult feasibility of the endovascular closure device within its instructions for use. Our FOPMAATT protocol allows immediate IVUS interrogation of the underlying etiology of the LGO and prompt correction.

**Fig:**
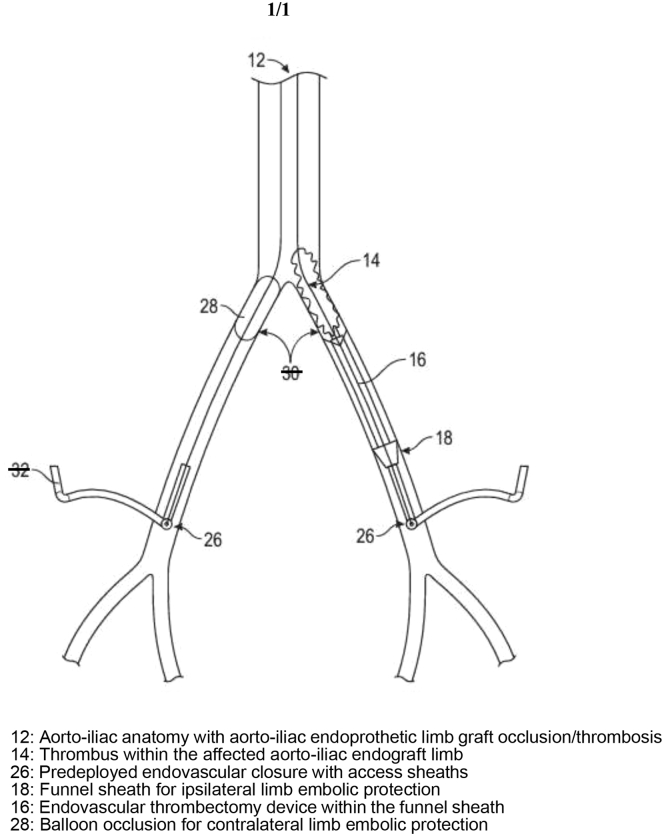
First-order percutaneous mechanical arterial advanced thrombectomy technology (FOPMAATT) protocol schematic.

Percutaneous bilateral common femoral artery access was obtained using ultrasound guidance and a micropuncture technique. We placed 5F sheaths into the bilateral common femoral artery access and then performed angiography to confirm the optimal access. Patients receive intravenous heparin for systemic anticoagulation intraoperatively, with an activated clotting time maintained at >230 seconds. Predeployment of one or two endovascular closure devices to the ipsilateral side and to the contralateral side if access sheath is >8F. In this protocol, the author used the Perclose ProGlide device (Abbott Vascular), which was deployed within ipsilateral common femoral arterial access with contralateral balloon occlusion for embolic protection in place. Successful predeployment of the Perclose endovascular closure device may dictate capability of endovascular approach. Additional care must be taken when manipulating the Perclose device into the thrombosed iliac limb. Contralateral balloon occlusion may help to decrease risk and prevent an embolic event to the contralateral limb. From the author's experience, a stiff glidewire can help to navigate within the acute and subacute thrombotic limb very well. Fluoroscopic guidance and visualization can be very helpful in monitoring Perclose tracking and deployment, taking care not to jam the Perclose catheter once the wire is removed. The blood flow side port may be very subtle and may not exhibit strong flow as with a normal deployment. The operator and their assistant must be aware and pay close attention to this factor.

Contralateral noncompliant balloon occlusion, size determined by preoperative contralateral endoprosthetic stent graft size, or using a compliant balloon occlusion in case of EVAR endoprosthetic stent graft, was placed in the proximal iliac limb for contralateral embolic protection. Sequential dilation to ipsilateral common femoral artery access was done to accommodate delivery of a 16F ClotTriever Sheath (Stryker-Inari Corporation). This funnel-shaped nitinol filtered sheath system was used to prevent thromboembolic complications downstream to the ipsilateral arterial system while thrombectomy was being performed (Fig).

Percutaneous transluminal mechanical thrombectomy of the thrombosed ipsilateral limb graft was performed through the ClotTriever Sheath. During thrombectomy procedure, FlowSaver (Stryker-Inari Corporation), a blood return system was used to return blood to the contralateral arterial access.

Once confirmed on angiography that the thrombus and occlusive pathology had been adequately removed, further investigation of the underlying cause of LGO/thrombosis was carried with IVUS examination. This investigation allowed LGO treatment of the underlying cause. A differential diagnosis of the potential etiology of LGO may include competitive flow owing to contralateral limb deployment or proximal migration, underexpansion of the ipsilateral limb graft owing to external compression from the iliac arterial pathology, or extensive aortoiliac pathology, which may cause disruption of inflow to the bilateral iliac limb.

After the underlying cause of occlusive disease had been treated, completion angiography and IVUS examination were again used to confirm the patency of the aortoiliac limb graft and adequate stent graft expansion. No thrombolytic agent was administered. All patients received subsequent inpatient care and were given dual antiplatelet therapy, smoking cessation counseling, and a high-dose statin. In some cases, for patients who continued tobacco use, rivaroxaban 2.5 mg twice daily was added to the standard medical therapy. All patients benefitted from outpatient clinical follow-up consisting of ankle-brachial index, computed tomography angiography, and outpatient clinical evaluation at 1-, 6-, 12-, and 24-months.

## Results

All five patients had common sets of comorbidities for those presenting with these conditions, most commonly hyperlipidemia (n = 5), type 2 diabetes (n = 3), hypertension (n = 3), and tobacco use (n = 3) (Table I and Table II). They were treated with aortoiliac stent placements between 2018 and 2022. Each patient suffered thrombosis or occlusion of their endoprosthetic stent graft limb. All patients were symptomatic with acute or subacute limb ischemia with Rutherford class 2b and required intervention to resolve the complication. These complications precipitated over a wide variety of time intervals from the initial intervention, with the earliest occurring after 1 week and the most remote complication appearing after 45 months. These complications typically presented with symptoms including pain, loss of temperature, and neurosensory deficit. Each procedure was successfully completed between 2021 and 2023. After resolution of the thrombus or occlusive burden, the percutaneous technique allowed us to optimally use IVUS examination to interrogate the underlying cause of the LGO/thrombosis. In case 1, a post-EVAR case, we found that the limb graft thrombosis was caused by a technical error at the stent graft implantation, which caused competitive flow and resulted in LGO. It was corrected by using IVUS-guided realignment of the ipsilateral aortoiliac limb graft. In case 2, IVUS examination visualized a significant atherosclerotic plaque burden to proximal landing zone of the iliac stent, which was corrected by proximal extension of the balloon expandable kissing stent.

**Table I:** Case details and their follow-up

	Case I	Case II	Case III	Case IV	Case V
Underlying vascular disease pathology	5.6 cm AAA	Occlusion of preexisting right iliac a. stent (from 2013)	Stenosis of B/L CIA, stenosis of prox. right EIA	Near occlusive disease of left CIA	Occlusion of left proximal CIA
Comorbidities	Tobacco use HLD COPD BPH Anxiety	HTN, HLD, DM2 CKD III COPD Cardiomyopathy	DM2 Obesity, OSA HTN, HLD, Tobacco, Psychiatric UTI at Presentation	Tobacco HLD Noncompliance	DM2, HTN, HLD Anxiety Depression History of Benzo OD, dementia Noncompliance
Primary intervention	8/19/2021 PEVAR w/IVUS	2/14/2022 Kissing PTA/stent in B/L CIA, PTA left EIA, IVUS	5/18/2022 B/L CIA stent, right EIA stent	May 4, 2021 Atherectomy PTA stent to left CIA	February 5, 2018 B/L CIA kissing stent 7/29/2019∗ Redo B/L CIA stent
Complication after primary intervention	Thrombosis of left iliac limb	Thrombosis reocclusion of right CIA stent	Occlusion of left CIA stent	Thrombosis of left iliac stent	Occlusion of left CIA stent, Severe stenosis of right CIA stent
Time between PRIMARY INTERVENTION and complication	11/30/2021 3 months	2 weeks	5/24/2022 1 week	8/16/2022 16 months	4/28/2023 45 months
Symptom of complication	Progressive claudication of left leg rest pain Neurosensory deficit	RLE claudication pain w/neurosensory deficit	Left leg weakness Rest pain Neurosensory deficit	Rest pain Neurosensory deficit	B/L leg weakness and claudication Left leg rest pain
Rutherford classification	2b	2 b	2b	2 b	2b
Secondary intervention	FOPMAATT 11/30/2021 No preoperative ABI owing to urgent presentation	FOPMAATT 3/17/2022 Preoperative ABI: R0.37 L0.86 (2/28/2022)	FOPMAATT March 6, 2022 Preoperative ABI: R0.67 L0.86 (November 4, 2022)	FOPMAATT 2/23/2023 Preoperative ABI: R0.99 L0.87 (1/23/2023)	FOPMAATT January 6, 2023 No preoperative ABI owing to urgent presentation
Hospital length of stay	1	1	Admitted 2 days before surgery for UTI, stayed for 4 days postoperative	1	1
One-month follow-up	ABI: N/A CTA: 5.2-cm AAA sac, patent aortobi-iliac stent graft	ABI: N/A CTA: N/A Additional ABI at 3 months: normal	ABI: R1.2 L1.1 (6/29/2022) CTA: normal, patent aortobi-iliac stent graft	ABI: N/A CTA: Patent B/L iliac artery Stable right retroperitoneal hematoma	ABI: N/A CTA: normal, patent aortobi-iliac stent graft
Six-month follow-up	ABI: R1.0 L1.0 (7/14/2022) CTA: 5.2-cm AAA, patent aortobi-iliac stent graft	ABI: R0.74 L1.0 (October 10, 2022) CTA: patent B/L stents, small dissection within right EIA but patent	ABI: R1.08 L1.07 (12/29/2022) CTA: normal, patent aortobi-iliac stent graft	ABI: N/A CTA: normal, patent aortobi-iliac stent graft	ABI: R1.08 L 0.92 (9/19/2023) CTA: normal, patent aortobi-iliac stent graft
Twelve-month follow-up	ABI: N/A CTA: 4.9 cm AAA, patent aortobi-iliac stent graft	ABI: N/A CTA: patent B/L, small dissection in right distal EIA	ABI: R1.2 L1.0 (8/23/2023) CTA: N/A	ABI: R 1.01 L 1.0 (3/13/2024) CTA: N/A	ABI: N/A CTA: N/A
Twenty-four-month follow-up	ABI: N/A CTA: 3.8 cm AAA sac, patent aortobi-iliac stent graft	ABI: R0.66 L1.1 (5/22/2024) CTA: N/A	ABI: N/A CTA: normal, patent aortobi-iliac stent graft ∗18-month follow-up	ABI: R1.02 L0.99 (1/29/2025) CTA: N/A	ABI: R0.81 L 051 (5/20/2025) CTA: N/A

*AAA,* Abdominal aortic aneurysm; *ABI,* ankle-brachial index; *B/L,* bilateral; *BPH,* benign prostate hypertrophy; *CIA,* common iliac artery; *CKD,* chronic kidney disease; *COPD,* chronic obstructive pulmonary disease; *CTA,* computed tomographic angiography; *DM2,* type 2 diabetes mellitus; *EIA,* external iliac artery; *FOPMAATT,* first-order percutaneous mechanical arterial advanced thrombectomy technology; *HLD,* hyperlipidemia; *HTN,* hypertension; *IVUS,* intravascular ultrasound; *L,* left; *N/A,* not available; *OSA,* obstructive sleep apnea; *PEVAR,* percutaneous endovascular aneurysm repair; *PTA,* percutaneous transluminal balloon angioplasty; *R,* right.

**Table II:** Preoperative and postoperative imaging

Case	Preoperative imaging	Postoperative imaging	Preoperative CTA	Postoperative angiogram
1				
2				
3				
4			Note: Not CTA	
5				

*CTA,* Computed tomographic angiography.

In case 3, the distal common iliac stent was externally compressed by heavily calcified atherosclerotic plaque, which was corrected by distal extension of the balloon-expandable stent. In case 4, IVUS examination revealed significant intimal hyperplasia causing in-stent restenosis and subsequent thrombosis; kissing balloon angioplasty has provided a good result along with optimal medical therapy. In case 5, on IVUS examination, we detected severe atherosclerotic disease at both the proximal and distal landing zones of the aortoiliac stent. It was corrected by realignment with kissing balloon-expandable stents proximally and left limb distal extension.

Postoperatively, patients underwent regular follow-ups at 1, 6, 12, and 24 months. We followed patients using computed tomographic angiography as the main modality in post-EVAR cases and the ankle-brachial index in conjunction with computed tomographic angiography in the other four cases with aortobi-iliac stent grafts (Table II). Cases 1 and 2 showed continuous improvement and remained symptom-free all the way through the 24-month interval. The aneurysm sac in case 1 continued to regress over the time course. Case 2, however, developed a small dissection in the contralateral distal external iliac artery that was noticed at the 6-month follow-up. Nonetheless, case 2 remains asymptomatic, and the repair was still patent bilaterally (Table II). We elected to not intervene and to continue clinical follow up. Cases 3, 4, and 5 remain free of vascular symptoms after 1 year.

Only one of the five patients had initial aortobi-iliac stent graft placement for AAA repair and four patients had initial aortoiliac stent graft placement for aortoiliac occlusive disease. All five patients experienced preoperative complications of thrombotic occlusion within a limb of aortoiliac stent graft. All five patients underwent FOPMAATT protocol management. We did not observe major perioperative complication. One of the 5 patients had a moderate hematoma at the contralateral access site with a 6F sheath, which appeared to be a failure of the endovascular closure device. The hematoma was managed conservatively, and the patient was able to ambulate the next day and be discharged home on postoperative day 1. None of the five patients received any pharmacological thrombolytic agent or blood product transfusion; therefore, no intensive care unit stay was required. Four of the five patients had a 1-day hospital length of stay; one patient had an extended length of stay owing to a preexisting urinary tract infection. The patient's underlying preoperative infection was managed by our hospitalist team and discharged home after day 4, once medically optimized. Secondary patency is 100% at 1, 3, 6, 12, and 24 months. No patients experienced a major adverse limb event, major adverse cardiac event, limb loss, or mortality in this series.

## Discussion

In this case series, the FOPMAATT technique resulted in minimal surgical length of stay, allowing patients to return home after 1 day. Case 3 was the only exception; the patient remained in the hospital for 4 days owing to a preexisting medical comorbidity (urinary tract infection) acquired preoperatively. A systematic review found that the average hospital stay for post-EVAR complication management was 4.95 days.^8^ No patient required intraoperative or perioperative blood transfusions. The same review found that the average amount of blood transfused for post-EVAR complication management was 0.83 U.^8^ More research needs to be done to determine if this trend persists in a larger sample size.

All five patients who underwent the FOPMAATT protocol procedure to treat the limb graft thrombotic occlusion have not reported any major complications. All five patients who had follow-up post FOPMAATT protocol (range, 24 to 36 months) were found to maintain patency of aortoiliac stent graft, consistent with current paradigms seeking value for the patient.^9^ Case 1 shows successful treatment of an LGO complication after EVAR. Cases 2 through 5 demonstrate successful treatment of an LGO complication after treatment for atherosclerotic occlusive disease.

## Conclusions

This case series reports five successful cases and their long-term outcomes. The FOPMAATT protocol effectively demonstrated low patient morbidity, and we observed no major adverse perioperative events. Of the five cases reported, we did not observe a blood loss significant enough to require transfusion, or a length of stay >1 day owing to any surgical cause. Additionally, we did not use adjuvant pharmacological thrombolytic therapy in the FOPMAATT protocol, which may decrease risk of perioperative bleeding and may eliminate the need for intensive care for these patients. Taken in conjunction with our previous results,^8^ we demonstrated long-term outcome benefits of the FOPMAATT protocol procedure. However, a larger number of cases should be analyzed for a better demonstration of the FOPMAATT protocol benefits in comparison with the conventional management of LGO in both post EVAR patients and post aortoiliac endoprosthetic stent graft for occlusive disease patients.

## Funding

None.

## Disclosures

None.
